# Deferoxamine treatment decreases amyloid fibrils and lowers iron mediated oxidative damage in ApoEFAD mice

**DOI:** 10.1002/alz.095418

**Published:** 2025-01-09

**Authors:** Max A Thorwald, Jose A Godoy‐Lugo, Amy Christensen, Christian J. Pike, Henry Jay Forman, Caleb E Finch

**Affiliations:** ^1^ USC Leonard Davis School of Gerontology, Los Angeles, CA USA; ^2^ University of Southern California, Los Angeles, CA USA; ^3^ University of California Merced, Merced, CA USA; ^4^ USC Dornsife College of Letters, Arts, and Sciences, Los Angeles, CA USA

## Abstract

**Background:**

Iron is implicated in Alzheimer’s disease (AD) and is bound to β‐amyloid (Ab) plaques. AD brains have increased 4‐hydroxynonenal (HNE) adducts, a lipid decomposition product bound to proteins originating from iron mediated lipid peroxidation. Increased brain iron may result from cerebral microbleeds which by nature are rich sources of iron. In EFAD aging, microbleeds arise before amyloid plaque formation, suggesting a role for microbleeds in plaque formation. We hypothesize that treatment with the iron chelator Deferoxamine (DFO) would reduce brain iron and attenuate oxidative damage from these microbleeds.

**Method:**

6‐month female EFAD mice were treated with DFO (10mg/kg/day) by diet (2 weeks; n = 9) or intraperitoneal injection (1 week; n = 11) and compared to untreated (n = 8). Cerebral cortex was assayed for soluble Aβ peptides, insoluble Aβ fibrils, and HNE by dot blot. Antioxidants were measured by Western blot or GPx assay (Cayman Chemical).

**Result:**

DFO in diet or by IP decreased insoluble fibrillar amyloid by 50% with corresponding 2‐fold increases in soluble Aβ monomers Aβ40 and ‐42. DFO reduced HNE levels by 50%; nitrotyrosine, a non‐iron dependent oxidation marker, remained unchanged. Protection from lipid oxidation is mediated by GPx4 or GSTA4, which DFO increased by 50%. These measures were complimented with 25% increases in total GPx activity by DFO.

**Conclusion:**

DFO solubilized fibrillar Aβ and increased Aβ monomers. Furthermore, DFO increased antioxidants critical to the protection against microbleed‐induced oxidative damage. These findings are substantiated by the decrease in HNE. Future studies will examine combination therapy with Aβ monoclonal antibodies which may liberate bound iron from Aβ plaques.

